# The neural basis of metacognitive ability

**DOI:** 10.1098/rstb.2011.0417

**Published:** 2012-05-19

**Authors:** Stephen M. Fleming, Raymond J. Dolan

**Affiliations:** 1 Center for Neural Science, New York University, 6 Washington Place, Room 809, New York, NY 10003, USA; 2 Wellcome Trust Centre for Neuroimaging, University College London, 12 Queen Square, London WC1N 3BG, UK

**Keywords:** metacognition, confidence, conflict, prefrontal cortex, functional magnetic resonance imaging, individual differences

## Abstract

Ability in various cognitive domains is often assessed by measuring task performance, such as the accuracy of a perceptual categorization. A similar analysis can be applied to metacognitive reports about a task to quantify the degree to which an individual is aware of his or her success or failure. Here, we review the psychological and neural underpinnings of metacognitive accuracy, drawing on research in memory and decision-making. These data show that metacognitive accuracy is dissociable from task performance and varies across individuals. Convergent evidence indicates that the function of the rostral and dorsal aspect of the lateral prefrontal cortex (PFC) is important for the accuracy of retrospective judgements of performance. In contrast, prospective judgements of performance may depend upon medial PFC. We close with a discussion of how metacognitive processes relate to concepts of cognitive control, and propose a neural synthesis in which dorsolateral and anterior prefrontal cortical subregions interact with interoceptive cortices (cingulate and insula) to promote accurate judgements of performance.

I am not yet able, as the Delphic inscription has it, to know myself, so it seems to me ridiculous, when I do not yet know that, to investigate irrelevant things.

Plato's *Phaedrus*, 229E

## 1. Introduction

The notion that accurate self-knowledge has value, and is something to strive for, has preoccupied thinkers since Socrates. But, as the quotation from Plato illustrates, self-knowledge is not always (or even often) evident, and at best tends to be a noisy and inaccurate impression of one's mental milieu [1]. Empirical work in the psychological sciences has thrown up counterintuitive examples of self-knowledge being confabulated, dissociated from reality or otherwise inaccurate [2,3]. To take one striking case, when decisions about facial attractiveness or supermarket goods are surreptitiously reversed, subjects are often unaware of these reversals, and go on to confabulate explanations of why they chose options they had in fact rejected [4,5]. Furthermore, self-assessments of personality and cognitive biases tend to be poorer than similar assessments applied to others, leading to an ‘introspection illusion’ [6]. Such subjective inaccuracy perhaps accounts for the demise of an introspectionist method in the late nineteenth century: if verbal reports vary from setting to setting, and can be contradicted from trial to trial, then what hope is there for an objective science of the subjective? [7].

The very notion that an individual can turn his or her mental faculties inward was considered logically incoherent by Comte, who thought it paradoxical that the mind might divide into two to permit self-observation [8]. We now understand the brain as a network of regions working in concert, and thus, it is perhaps unsurprising that one set of regions (such as the prefrontal cortex: PFC) might process, hierarchically, information arising from lower levels (such as primary sensory regions). Indeed, several recent models of local and large-scale brain function rely on hierarchy as a principal organizing factor [9,10]. That self-knowledge, and its accuracy, is under neural control is supported by mounting evidence in the neuropsychological literature, some of which will be reviewed later in this article. For example, in cases of traumatic injury to the frontal lobes, individuals may have deficits in self-knowledge of altered cognition and personality, as measured by the discrepancy between reports from the patient and family members [11]. Such studies have focused on alterations in self-related, or autonoetic, metacognition [12], but analogous discrepancies can be measured in assessments of task performance in healthy individuals.

**Figure 1.**
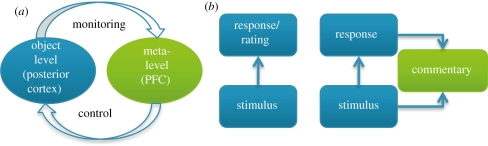
(*a*) A schematic adapted from Shimamura [16] showing how the levels of Nelson and Narens' cognitive psychology model of metacognition can be naturally mapped onto a hierarchical brain structure. (*b*) The left panel shows a first-order process, such as a simple visual discrimination, that may occur in the absence of metacognitive report. The right panel shows the same discrimination, this time with the information available for a second-order commentary about the decision.

By focusing on self-reports about memory performance—metacognitive reports—Flavell provided a systematic framework for the study of self-knowledge in healthy individuals [13]. Here, the metacognitive report is treated as an object of study in its own right, and the accuracy of such reports (as dissociated from accuracy, or performance, on the task itself) provide an empirical scaffold upon which to build studies of self-knowledge [14,15]. An influential model of metacognition was developed to account for behavioural dissociations between the ‘object’ level—cognition, or, more correctly, task performance—and the ‘meta’ level, conceptualized as both monitoring and controlling the object level (figure 1; [17]). This approach shares similarities with an influential model of executive function [18]. The two-level framework has been extended to study monitoring of perception [19,20], decision-making [21,22], sense of agency [23] and learning [24]. To the extent that the meta level imperfectly monitors the object level, self-reports about cognition will be inaccurate, perhaps manifesting as a lack of awareness of the object level [25].

Despite progress in the definition and measurement of metacognition, the psychological and neural underpinnings of metacognitive accuracy remain ill understood [16,26]. In this paper, we review different approaches to eliciting metacognitive reports and quantifying their accuracy, and consider psychological and computational explanations for dissociations between metacognitive accuracy and task performance. We go on to consider recent studies that apply convergent neuroscience methodologies—functional and structural magnetic resonance imaging (MRI), transcranial magnetic stimulation (TMS) and neuropsychological approaches—to reveal cortical substrates mediating differences in metacognitive accuracy both between and within individuals. We end with a discussion of how metacognitive processes relate to neuroscientific notions of cognitive control, and propose a synthesis wherein dorsolateral and anterior prefrontal cortical subregions interact with interoceptive cortices (cingulate and insula) to promote metacognitive accuracy.

## 2. Measurement of metacognition

There are several flavours of metacognitive report, but all share the elicitation of subjective beliefs about cognition—how much do I know (viz. what can I report) about ongoing task performance? In this section, we review the behavioural methods available to the researcher interested in metacognition, focusing primarily on measures employed in the cognitive neuroscience studies that are discussed in subsequent sections.

A first distinction is that judgements can either be prospective, occurring prior to performance of a task, or retrospective, occurring after task completion (table 1). In metamemory research, prospective judgements include feelings of knowing (FOK) and judgements of learning (JOL). A JOL elicits a belief during learning about how successful recall will be for a particular item on subsequent testing [27]. In contrast, an FOK is a judgement about a different aspect of memory, namely that of knowing the answer to a particular question despite being unable to explicitly recall it [28]. FOKs are usually studied by first asking the participants to recall answers to general knowledge questions, and, for answers they cannot recall, to predict whether they might be able to recognize the answer from a list of alternatives. Related to FOKs are tip-of-the-tongue states, in which an item cannot be recalled despite a feeling that retrieval is possible [29].

**Table 1.** Summary of metacognitive measures classified by domain and time of elicitation. We note that a more general class of prospective judgements is also possible that refers to cognitive abilities not tied to a particular task.

timing	object-level domain		
	memory	decision-making	sensory
prospective	judgement of learning; feeling of knowing	performance estimate	n.a.
retrospective	confidence	confidence, wager	visibility rating, confidence

Retrospective reports can be similarly elicited by asking the subject to give an additional report or commentary over and above their initial forced-choice response. For example, Peirce & Jastrow [30] asked observers to rate their degree of confidence in a perceptual judgement using the following scale:

0 denoted absence of any preference for one answer over its opposite, so that it seemed nonsensical to answer at all. ‘1’ denoted a distinct leaning to one alternative. ‘2’ denoted some little confidence of being right. ‘3’ denoted as strong a confidence as one would have about such sensations.

Since this seminal work, asking for confidence-in-accuracy has become a standard tool for eliciting judgements of performance in a variety of settings [24,31]. One potential problem with eliciting subjective confidence is that of reliability: why should the subject be motivated to reveal his or her true confidence, when there is little incentive to do so [32]? In addition, the necessarily subjective instructions given when eliciting reports of confidence preclude the use of these measures in non-human animal species. To address these concerns, Kunimoto and colleagues introduced wagers contingent on the correctness of the decision as an intuitive measure of retrospective confidence [33,34]. In the simplest form of post-decision wagering (PDW), a participant is asked to gamble on whether their response was correct. If the decision is correct, the wager amount is kept; if it is incorrect, the amount is lost. The size of the chosen gamble is assumed to reflect a subject's confidence in his or her decision. In the same spirit as PDW, the Lottery Rule aims to elicit true underlying decision confidence [35], and is similar to the Becker–DeGroot–Marschak procedure used to elicit item values in behavioural economics [36].

Once a metacognitive judgement is elicited, how might we assess its accuracy? Again, several, often complementary, methods are available. Metacognitive accuracy is defined by how closely metacognitive judgements track ongoing task performance. Crucially, therefore, all measures require that an independent measure of the object level—task performance—is acquired, in order to quantify the relationship between the meta and object levels (figure 1). For example, after asking for an FOK judgement, we might assess whether the proportion of times a participant is indeed able to recognize the correct, but hitherto unrecalled, item from a list of alternatives. Then, by plotting the strength of the JOL or FOK against objective memory performance (actual recall success for JOLs, and recognition performance for FOKs), a measure of metacognitive accuracy can be derived from the associated correlation score [15]. Similar confidence-accuracy correlations can be computed for retrospective confidence judgements. If the metacognitive report bears some relation to task performance, then these correlation coefficients will be significantly non-zero [37].

**Figure 2.**
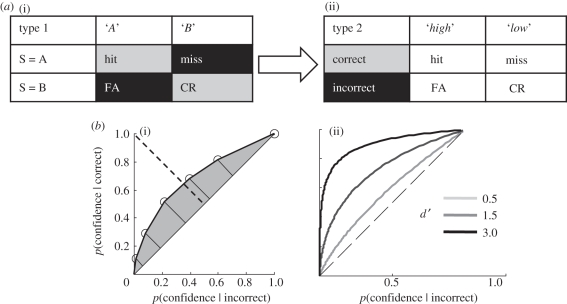
(*a*) Contingency tables for (i) type 1 SDT, and (ii) type 2 SDT. Rows correspond to objective states of the world; columns correspond to subjects' reports about the world; FA, false alarm; CR, correct rejection. In the type 2 table, ‘high’ and ‘low’ refer to decision confidence. The linking arrow and colour scheme indicates that ‘correct’ and ‘incorrect’ states of the world for the type 2 analysis are derived from averaging particular type 1 outcomes. (*b*) (i) Example of a type 2 receiver operating characteristic (ROC) function for a single subject in a perceptual decision task where performance is held constant using a staircase procedure. The shaded area indicates the strength of the relationship between performance and confidence. (ii) Theoretical type 2 ROC functions for different levels of type 1 *d*′ (assuming neutral type 1 response criteria) demonstrating that metacognitive accuracy is predicted to increase as task performance increases.

A related approach quantifies the accuracy of metacognitive assessments using the logic of signal detection theory (SDT), which assesses how faithfully an organism separates signal from noise [38,39]. In standard applications of SDT (type 1), sensitivity is defined by how well an observer can discriminate an objective state of the world (e.g. the presence or absence of a stimulus; figure 2*a*). By applying similar logic to metacognitive reports, the objective state of the world becomes the subject's trial-by-trial task performance (correct or incorrect; figure 2*a*) and the subjective report is now a judgement of that performance [40,41]. An advantage of the SDT approach is that it dissociates bias from sensitivity: in other words, measures of metacognitive accuracy are relatively unaffected by an observer's overall tendency to use higher or lower confidence ratings (figure 2*b*; although see [42,43]). Further, it naturally connects a process-level characterization of the relationship between the object (type 1) and meta level (type 2) to measures of behaviour, and this relationship can be taken into account to provide an unbiased measure of metacognitive accuracy [44]. This generative aspect of SDT will be discussed further in a following section.

Before closing our discussion on measures of metacognition, we note that a separate line of research has assessed the extent to which humans and other species use, or represent, uncertainty about the consequences of their actions to optimize decision-making (see [45,46] for reviews). To highlight one example, Barthelme & Mamassian showed that when human observers are allowed to choose between pairs of visual stimuli upon which to carry out a task, they systematically chose the less uncertain, thus improving their performance [47]. Related work has demonstrated that subjects use knowledge of uncertainty to optimally bias decision-making in perceptual [48,49] and motor [50] tasks, and that species as diverse as dolphins, pigeons and monkeys can use an ‘opt-out’ response to improve their reward rate when decisions are uncertain [51]. Recent single-neuron recording studies have begun to outline candidate mechanisms for a representation of uncertainty in the decision system [52,53]. However, and crucially for the purposes of the present paper, use-of-uncertainty measures do not dissociate metacognition from task performance on a trial-by-trial basis, and thus cannot be used to study mechanisms underlying beliefs about performance. For example, on each trial of the ‘opt-out’ paradigm, the animal either chooses to complete the task, or opt-out. On trials where the animal opts-out (uses a ‘metacognitive’ response), we are unable to measure performance, as no task is completed. On trials where the animal does not opt-out, performance measures are all we have. Thus, measures of metacognitive accuracy cannot be computed based on pairwise correlations between the two response types [54].

## 3. Psychological determinants of metacognitive accuracy

In healthy individuals, metacognitive judgements are usually predictive of subsequent or past task performance [55]. What, then, underlies this ability to know that we know? On a direct-access view, metamemorial judgements are based upon a survey of memory contents, and thus draw upon the same information as a subsequent recognition or recall phase [28]. In contrast, inferential accounts suggest that JOL, FOK and confidence judgements draw upon various mnemonic cues that may only be partially related to the target [56] (see [57] for a review). Such cues include the fluency or ease with which information is processed [58,59], the accessibility or relatedness of cue information to the target [60] and, for retrospective confidence judgements, the speed of a previous decision [17,61]. Because the available cues may only be indirectly related to the target, inferential accounts naturally accommodate dissociations between memory performance and metacognitive accuracy; in contrast, direct-access accounts predict a tight relationship between subjective and objective indices of knowledge.

**Figure 3.**
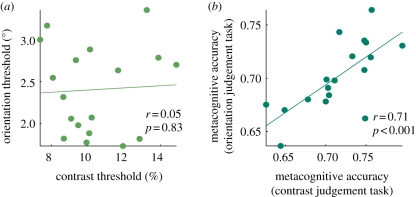
Data from a visual decision task demonstrating a dissociation of metacognitive accuracy from task performance. Subjects made a visual decision (either an orientation or contrast judgement) and then provided a retrospective confidence rating. A measure of metacognitive accuracy was derived from these ratings by calculating the area under the type 2 ROC function. Performance on the orientation judgement task did not predict task performance on the contrast judgement task (*a*). However, metacognitive accuracy was strongly correlated between tasks (*b*), suggesting that it is both independent of task performance and stable within individuals. Reproduced with permission from Song *et al*. [70].

A complementary perspective on the antecedents of metacognitive reports is provided by type 2 SDT. Consider a perceptual decision task where post-decision wagers are elicited to tap knowledge of task performance. Optimal wagering behaviour requires computing the conditional probability of being correct given a previous choice [*p*(correct|choice)] to decide whether to wager high or low. There are various proposals as to how this might be achieved [43,62]. In an echo of direct-access accounts of metamemory discussed above, most involve tracking the strength of the underlying evidence entering into the choice. Galvin and colleagues [41] showed that the conditional probability of being correct or incorrect for a given decision signal is a simple linear transformation of type 1 probability distributions. Similarly, in a dynamic situation, Vickers [31] proposed that decision confidence could be derived from the absolute distance between the winning and losing integrators in an evidence accumulation framework (see also [52]). Confidence, therefore, is equated with the difficulty of the decision in these approaches [63,64]. Two corollaries arise from this ‘direct translation hypothesis’ [65]. First, given that confidence is equated with choice probability (as derived from information governing choice), direct-translation approaches cannot accommodate dissociations between the object and meta level. Second, if both performance and metacognitive judgements draw upon the same information, metacognitive accuracy or the ability to discriminate correct from incorrect decisions, always increases as task performance itself increases. Importantly, both these hypotheses have been empirically falsified: for the same level of task performance, judgement confidence may differ considerably between conditions [66–68], and, when performance is held constant using a staircase procedure, metacognitive accuracy varies across individuals [21], and can be dissociated from performance through pharmacological [69], neural [20] and task-based [70] manipulations (figure 3).

Empirical dissociations between first-order and second-order components of decision-making have prompted a search for models that can accommodate such findings [71]. Recent models have been couched in an ‘evidence accumulation’ framework, in which samples of data are accumulated over time in order to model the temporal evolution of a decision [19,72,73]. Del Cul *et al*. [19] proposed a dual-route evidence accumulation framework in which evidence for behaviour (a forced-choice report of stimulus identity) and evidence for subjective report (visibility) were accumulated separately. The fit of this model could account for the observed decoupling of subjective reports from performance in patients with damage to the PFC (see the study of Maniscalco & Lau [74] for an alternative account). In a related approach, Pleskac & Busemeyer [72] devised an evidence accumulation scheme that could account for a wide range of empirical regularities governing the relationship between choice and confidence ratings. The solution here was to allow accumulation to continue beyond the time at which the first-order decision is made. The same noisy accumulator is then accessed to form the confidence judgement at a later timepoint. Interestingly, this model makes strong predictions about post-decision neural activity in the parietal and frontal cortices previously associated with pre-decision evidence accumulation [75], and recent developments of PDW methods in non-human primates may allow this and related hypotheses to be tested [76].

Despite being dissociable, metacognitive accuracy does generally scale with task performance [33,77–80]. Note that this regularity differs conceptually from the fact that trial-by-trial judgements of confidence tend to correlate with performance; such scaling is, after all, what measures of metacognitive accuracy attempt to capture. Instead, it is the fact that, between sessions, or individuals, metacognitive accuracy itself covaries with performance on the task (figure 2*b*). A tied relationship between performance and metacognition presents a particular problem for studies of the neural correlates of metacognitive ability: how are we to disentangle brain systems involved in metacognition from those involved in performing the task itself (cf. [81])? In the following section, we keep this confound of performance in mind, and consider the extent to which it is addressed by studies of the neural basis of metacognitive accuracy.

## 4. Neural basis of metacognitive accuracy

### (a) Studies of metamemory

Initial evidence regarding the neural basis of metacognition was obtained from neuropsychological cases [82]. Hirst and colleagues suggested that metamemory might be impaired in patients with Korsakoff's syndrome, a neurological disorder characterized by severe anterograde amnesia that occurs as a result of chronic alcohol abuse and nutritional deficiency [83]. Structural brain changes in Korsakoff's include increases in cerebrospinal fluid and severe volume loss in the orbitofrontal cortices and thalamus [84]. Shimamura & Squire [85] found that Korsakoff's patients have a selective impairment in the accuracy of FOK judgements compared with an amnesic control group, despite being equated on recognition memory performance. These findings suggested that metamemory impairment is due to damage in brain regions other than medial temporal lobe and diencephalic midline structures associated with amnesia. In line with this hypothesis, subsequent studies found that non-amnesic patients with frontal lobe damage also exhibit poor metamemory accuracy (e.g. [86]; see [87] for a review).

While implicating frontal lobe structures in metacognitive accuracy, these early studies lacked anatomical specificity. Using lesion overlap measurements, Schnyer and colleagues found that damage to the right ventromedial prefrontal cortex (VMPFC) was associated with decreased FOK accuracy but intact confidence judgements, suggesting a possible dissociation between brain systems supporting different classes of metamemorial judgements [88] (table 1). Patients in Schnyer *et al.*'s study also showed deficits in memory performance, but impairment in FOK accuracy could not be explained by these changes in performance alone. In support of a selective role for medial PFC in FOK judgements, patients with lesion overlap in the dorsal anterior cingulate cortex (ACC) who were matched in recognition performance to a control group showed a selective FOK deficit, despite intact confidence judgements [79]. The reverse dissociation was reported by Pannu *et al.* [89], who found that deficits in retrospective confidence judgements were predominantly associated with lateral frontal lesions. As we discuss below, together this evidence suggests that prospective judgements are supported by medial PFC function, whereas retrospective judgements depend on lateral PFC.

Complementary functional brain imaging studies have shown that regions in the medial and lateral PFC are active during metamemorial judgements, with activity in PFC modulated by both prospective and retrospective confidence judgements [90–94]. VMPFC (peak Montreal Neurological Institute coordinate: −3, 30, −18) showed greater activity during accurate FOK judgements, and increased connectivity with medial temporal lobe memory structures in the FOK condition compared with a low-level control task [95]. Complementing this work, individual differences in metacognitive accuracy for prospective JOLs correlated with VMPFC activity (peak: −11, 42, −26) on accurate, but not inaccurate, prediction trials [78]; these differences were not explained by individual differences in memory performance.

### (b) Retrospective confidence judgements in psychophysics

**Figure 4.**
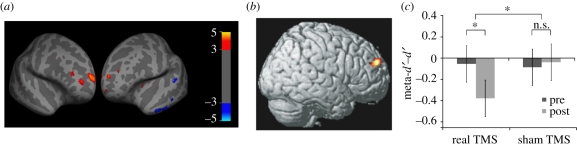
Convergent evidence for a role of rostrolateral PFC in metacognitive accuracy. (*a*) Across individuals, grey matter volume in rlPFC was found to positively correlate (hot colours) with metacognitive accuracy (type 2 ROC area) after controlling for differences in task performance [21]. (*b*) In a complementary study, BOLD signal in right posterior-lateral BA10 was positively correlated with metacognitive accuracy (gamma) but not differences in task performance [96]. (*c*) The necessity of lateral PFC for metacognitive accuracy was confirmed by combining TMS with SDT: following repetitive TMS to bilateral dlPFC, subjects exhibited reduced meta-*d*′ (the type 2 *d*′ expected from a given level of type 1 sensitivity) despite intact task performance [20]. Panels reproduced with permission from [21,96,20].

Other studies have begun to harness the methods of psychophysics to tightly clamp or adjust for differences in performance while simultaneously studying metacognition and its neural substrates (figure 4).

As an example of this approach, Lau and Passingham matched performance between two visual masking conditions, but found differences in threshold for metacognitive commentaries about the stimulus (‘seen’ responses) that were associated with activity in left dorsolateral PFC [67] (dlPFC; peak: −46, 48, 14). Confirming a causal role for PFC in subjective report threshold, patients with lesions to rostrolateral prefrontal cortex (rlPFC, BA10) have an increased threshold for producing metacognitive commentaries about a stimulus compared with controls, despite objective performance being matched between groups [19]. The peak correlation between lesion and decrease in subjective report threshold was seen in left BA10 (peak: −32, 54, −6).

Taking an individual differences approach, Fleming *et al.* [21] constrained perceptual decision performance to be near-threshold (71%) through use of a staircase procedure, while collecting retrospective confidence ratings. Considerable variation in metacognitive accuracy (using type 2 SDT analysis) was found despite task performance remaining constant across individuals. Through use of structural brain imaging, this variance in metacognitive accuracy was shown to positively correlate with grey matter volume in right rlPFC (BA10; peak: 24, 65, 18; figure 4*a*), and greater metacognitive accuracy was associated with increased white matter integrity (fractional anisotropy) in a region of the corpus callosum known to project to the rlPFC [97]. Such findings are consistent with individual differences in localized brain structure affecting a region's functional properties [98]. In a complementary study using functional MRI, subjects performed a visual working-memory test and provided retrospective confidence ratings. Metacognitive accuracy as determined by the gamma statistic correlated with the level of activity in right posterior-lateral BA10 [96] (peak: 16, 56, 28), despite being uncorrelated with task performance (figure 4*b*).

While correlational analyses can reveal candidate brain regions mediating metacognitive accuracy, confirmation of their necessity ultimately requires intervention studies. By applying repetitive TMS to temporarily inactivate bilateral dlPFC, Rounis *et al.* [20] selectively decreased metacognitive accuracy while leaving performance on a perceptual task unaffected. Further, by explicitly modelling the link between type 1 and type 2 responses [44], they were able to show that dlPFC TMS decreased metacognitive accuracy below that expected from a direct-translation account alone (figure 4*c*). Taken together, these studies provide convergent evidence that rostrolateral aspects of PFC (BA10/46) play a mediating role in the accuracy of retrospective commentaries.

A role for rlPFC in metacognition is consistent with its anatomical position at the top of the cognitive hierarchy, receiving information from other prefrontal cortical regions, cingulate and anterior temporal cortex [99]. Further, compared with non-human primates, rlPFC has a sparser spatial organization that may support greater interconnectivity [100]. The contribution of rlPFC to metacognitive commentary may be to represent task uncertainty in a format suitable for communication to others, consistent with activation here being associated with evaluating self-generated information [101,102], and attention to internal representations [103]. Such a conclusion is supported by recent evidence from structural brain imaging that ‘reality monitoring’ and metacognitive accuracy share a common neural substrate in anterior PFC [104]. In contrast, dlPFC may maintain information about a previous decision, consistent with its role in working memory [105,106]. However, in comparison with, for example, parietal cortex [107], reliable cytoarchitectonic boundaries are not yet established for human rlPFC [108]. Indeed, activations ascribed to either lateral rlPFC or dlPFC in this review cluster around a transition zone between BA10 and BA46 [96,109]; thus, it is unclear whether they arise from a single functional region, or multiple subregions subserving different functions. Single-subject analyses [110] may aid in solving this puzzle.

### (c) Nature of individual differences

Harnessing individual differences can provide leverage on the neural correlates of metacognitive accuracy [21,78,96]. Such studies implicitly assume intrapersonal stability of metacognitive capacity. However, in the metamemory literature, evidence for a stable metacognitive ability is surprisingly weak [111,112]. Given the interdependence of metacognition and performance discussed above, one explanation for this null result might be methodological in nature, as a performance-confidence relationship is naturally harder to quantify than performance itself. A similar line of thought led Keleman *et al.* to speculate that ‘stable metacognitive performance might be detected using very large numbers of trials’ [112]. In support of this view, Fleming *et al.* showed good split-half reliability (*r* = 0.69) in a perceptual decision task with hundreds of trials [21], and metacognitive accuracy has been shown to be stable across two perceptual tasks (*r* = 0.71), despite performance itself being uncorrelated (*r* = 0.05; figure 3) [70]. An important unanswered question is whether metacognitive accuracy is stable across domain (e.g. memory and decision-making), as might be predicted by their overlapping neural substrates [113].

### (d) Summary

There is now considerable evidence that damage to the PFC selectively affects the accuracy of metacognitive reports while leaving task performance relatively intact. Intriguingly, there is some evidence for a lateral–medial separation between neural systems supporting retrospective confidence judgements and prospective judgements of performance, respectively. The role of ventromedial PFC in prospective judgements of performance may be explained by its strong connections with medial temporal lobe memory structures and its role in imagination of the future [114,115]. In contrast, the role of anterior and dorsolateral PFC in retrospective judgements of confidence may be more closely aligned to that of a performance monitor, integrating and maintaining information pertaining to the immediately preceding decision to facilitate accurate metacognitive commentary. In the next section, we focus in greater detail on performance-monitoring functions to illustrate connections between metacognition and a separate but substantial literature on the neuroscience of cognitive control.

## 5. Relationship between metacognition and cognitive control

An influential suggestion is that decision-making systems should be sensitive to the current level of conflict between possible responses to mobilize additional ‘cognitive control’ resources in an adaptive fashion [116]. Activity in ACC and anterior insula is increased during heightened response conflict (see [117,118] for reviews), whereas lateral PFC activity correlates with behavioural adjustments, such as increased caution, following high-conflict trials [119,120]. Further, the ACC is suggested to recruit lateral PFC to increase levels of control when conflict occurs [117]. This proposal for a cognitive control loop shares obvious similarities with concepts of monitoring and control in metacognition research (figure 1); indeed, a previous review proposed metacognition might be commensurate with cognitive control [121]. However, such a view would predict that *any* system with the capacity for monitoring and control has metacognitive representations, which is not usually held to be the case. Instead, philosophers have discussed and debated two ‘levels’ of metacognition [122]: one involving declarative (conscious) meta-representation [123]; the other low-level, based on non-verbal epistemic feelings of uncertainty [124,125]. For present purposes, we consider monitoring processes as metacognitive to the extent they are consciously reportable, and thus available for deployment outside of a ‘closed-loop’ optimization of the task at hand (see also [126]). Such reports can be empirically dissociated from monitoring and control: for example, skilled typists show subtle post-error adjustments in the absence of awareness, and yet accept blame for errors that are surreptitiously inserted by the experimenters on the screen [127]. Interestingly, subjective effects of heightened decision conflict may themselves be reportable in the absence of awareness of antecedents of this conflict [128], and thus it is not always simple to decide whether performance monitoring involves meta-representation.

What might govern the accessibility of performance-monitoring information to awareness? We suggest that rlPFC is particularly important for the representation of information pertaining to a previous decision in a globally accessible frame of reference. In a direct comparison of confidence judgements following mnemonic and perceptual decisions, both ACC and right dlPFC activity increased with decreasing confidence [113]; however, only right dlPFC encoded confidence independent of changes in reaction time, leading the authors to suggest that while ACC responds to online decision conflict, dlPFC activity underlies the selection of metacognitive responses. Furthermore, a recent study found that activity in rlPFC both increases during metacognitive reports and correlates with reported confidence [109]. Thus, the accuracy of metacognitive commentaries, as dissociated from adjustments in performance, might be governed by the fidelity with which rlPFC integrates and maintains information from cingulate and insula involved in online adjustments in task performance, consistent with reciprocal anatomical connections between these regions [129].

If only a subset of nodes in this network is present, one might find effective performance monitoring in the absence of metacognition. This pattern of results was observed in a patient with a large left prefrontal cortical lesion, who displayed intact performance adjustments in the Stroop task, without being able to report changes in the subjective sense of effort while performing the task [130]. As the patient displayed intact conflict-related N2 event-related potential responses during the Stroop task, the authors suggested that (implicit) monitoring and control is maintained by an intact right ACC, while a subjective feeling of effort would normally be mediated by the damaged lateral PFC. Such a conclusion is supported by recent evidence that lateral PFC activity is higher in subjects with a strong tendency to avoid cognitively demanding decisions [131]. Importantly for our hypothesis, if lateral PFC receives input from non-conscious monitoring loops, the reverse dissociation would not be predicted: we might be able to control objects we cannot report, but should not be able report upon objects we cannot (cognitively) control.

The respective roles of nodes in this network remain to be determined, but there is initial evidence for division of labour. TMS to dlPFC impairs metacognition following correct but not incorrect decisions, suggesting a role in representing confidence rather than monitoring for errors [20]. In contrast, reporting of response errors has been linked to the error-related positivity [132] with a possible source in insula cortex [118]. Indeed, accurate metacognitive commentaries about *performance* require access to information about both beliefs and responses. For example, just after hitting a shot in tennis, you might have high confidence (low uncertainty) that the spot you chose to aim at is out of reach of your opponent (your belief), but low confidence in correctly executing the shot (your response). Thus, for commentaries to integrate information both about a belief and response, the ‘frame of reference’ in which information is encoded is crucial. If information is maintained in segregated sensorimotor loops, performance adjustments could be made based on deviations from an expected trajectory without this information being more generally available for, say, verbal report. It remains an open question as to the extent to which decision-making relies on ‘embodied’ or domain-general circuitry [133], but a role for the PFC in the abstract encoding of decision-related information, independent of response modality, has been found using fMRI conjunction analyses [134,135]. It will be of interest to test whether this same activity is involved in metacognition.

## 6. Conclusions

Cognitive psychology has developed a rich theoretical framework and empirical tools for studying self-assessments of cognition. A crucial variable of interest is the accuracy of metacognitive reports with respect to their object-level targets: in other words, how well do we know our own minds? We now understand metacognition to be under segregated neural control, a conclusion that might have surprised Comte, and one that runs counter to an intuition that we have veridical access to the accuracy of our perceptions, memories and decisions. A detailed, and eventually mechanistic, account of metacognition at the neural level is a necessary first step to understanding the failures of metacognition that occur following brain damage [87] and psychiatric disorder [136]. In this paper, we summarized a variety of behavioural approaches for measuring the accuracy of metacognitive assessments, and reviewed the possible neural substrates of metacognitive accuracy in humans. We conclude that there are potentially separable brain systems for prospective and retrospective judgements of performance, and our synthesis of recent neuropsychological and brain imaging findings implicates the rostrolateral PFC as crucial in mediating retrospective judgements of cognition. In this model, the rostrolateral PFC receives input from interoceptive cortex involved in ‘closed-loop’ monitoring and control, generating a metacognitive representation of the state of the system that can be deployed or reported outside of the current task at hand.

We close with a number of open questions we hope will be addressed by future studies:

— To what extent does metacognitive accuracy (and its associated neural correlates) generalize across different object-level domains?
— To what extent does metacognition rely on abstract (response-independent) decision variables?
— Are the neural correlates of error-monitoring and confidence separable [71]?
— Do dlPFC (∼BA46) and rlPFC (∼BA10) make differential contributions to metacognition?
— If task performance can be monitored and corrected in the absence of metacognitive report, what is the functional role of metacognitive (in)accuracy?

## References

[1] Carruthers P. 2011 The opacity of mind: an integrative theory of self-knowledge. New York, NY: Oxford University Press

[2] Nisbett R. E. Wilson T. D. 1977 Telling more than we can know: verbal reports on mental processes. Psychol. Rev. 84, 231 10.1037/0033-295X.84.3.231 (doi:10.1037/0033-295X.84.3.231)

[3] Wilson T. D. Dunn E. W. 2004 Self-knowledge: its limits, value, and potential for improvement. Ann. Rev. Psychol. 55, 493–518 10.1146/annurev.psych.55.090902.141954 (doi:10.1146/annurev.psych.55.090902.141954) 14744224

[4] Johansson P. Hall L. Sikström S. Olsson A. 2005 Failure to detect mismatches between intention and outcome in a simple decision task. Science 310, 116–119 10.1126/science.1111709 (doi:10.1126/science.1111709) 16210542

[5] Hall L. Johansson P. Tärning B. Sikström S. Deutgen T. 2010 Magic at the marketplace: choice blindness for the taste of jam and the smell of tea. Cognition 117, 54–61 10.1016/j.cognition.2010.06.010 (doi:10.1016/j.cognition.2010.06.010) 20637455

[6] Pronin E. 2007 Perception and misperception of bias in human judgment. Trends Cogn. Sci. 11, 37–43 10.1016/j.tics.2006.11.001 (doi:10.1016/j.tics.2006.11.001) 17129749

[7] Boring E. 1953 A history of introspection. Psychol. Bull. 50, 169–189 10.1037/h0090793 (doi:10.1037/h0090793) 13056096

[8] James W. 1950 The principles of psychology, vol. 1 New York, NY: Dover Publications

[9] Friston K. 2005 A theory of cortical responses. Phil. Trans. R. Soc. B 360, 815–836 10.1098/rstb.2005.1622 (doi:10.1098/rstb.2005.1622) 15937014 PMC1569488

[10] Koechlin E. Hyafil A. 2007 Anterior prefrontal function and the limits of human decision-making. Science 318, 594–598 10.1126/science.1142995 (doi:10.1126/science.1142995) 17962551

[11] Schmitz T. W. Rowley H. A. Kawahara T. N. Johnson S. C. 2006 Neural correlates of self-evaluative accuracy after traumatic brain injury. Neuropsychologia 44, 762–773 10.1016/j.neuropsychologia.2005.07.012 (doi:10.1016/j.neuropsychologia.2005.07.012) 16154166

[12] Metcalfe J. Van Snellenberg J. X. DeRosse P. Balsam P. Malhotra A. K. 2012 Judgments of agency in schizophrenia: an impairment in autonoetic metacognition. Phil. Trans. R. Soc. B 367, 1391–1400 10.1098/rstb.2012.0006 (doi:10.1098/rstb.2012.0006) PMC3318770 22492755

[13] Flavell J. 1979 Metacognition and cognitive monitoring: a new area of cognitive-developmental inquiry. Am. Psychol. 34, 906–911 10.1037/0003-066X.34.10.906 (doi:10.1037/0003-066X.34.10.906)

[14] Ericsson K. Simon H. 1980 Verbal reports as data. Psychol. Rev. 87, 215–251 10.1037/0033-295X.87.3.215 (doi:10.1037/0033-295X.87.3.215)

[15] Nelson T. 1984 A comparison of current measures of the accuracy of feeling-of-knowing predictions. Psychol. Bull. 95, 109–133 10.1037/0033-2909.95.1.109 (doi:10.1037/0033-2909.95.1.109) 6544431

[16] Shimamura A. P. 2008 A neurocognitive approach to metacognitive monitoring and control. In Handbook of memory and metamemory: essays in honor of Thomas O. Nelson (eds Dunlosky J. Bjork R.), pp. 373–390 New York, NY: Psychology Press

[17] Nelson T. O. Narens L. 1990 Metamemory: a theoretical framework and new findings. Psychol. Learn. Motivation: Adv. Res. Theory 26, 125–173 10.1016/S0079-7421(08)60053-5 (doi:10.1016/S0079-7421(08)60053-5)

[18] Shallice T. Burgess P. 1996 The domain of supervisory processes and temporal organization of behaviour. Phil. Trans. R. Soc. B 351, 1405–1411; discussion 1411–1412 10.1098/rstb.1996.0124 (doi:10.1098/rstb.1996.0124) 8941952

[19] Del Cul A. Dehaene S. Reyes P. Bravo E. Slachevsky A. 2009 Causal role of prefrontal cortex in the threshold for access to consciousness. Brain 132, 2531 10.1093/brain/awp111 (doi:10.1093/brain/awp111) 19433438

[20] Rounis E. Maniscalco B. Rothwell J. Passingham R. Lau H. 2010 Theta-burst transcranial magnetic stimulation to the prefrontal cortex impairs metacognitive visual awareness. Cogn. Neurosci. 1, 165–175 10.1080/17588921003632529 (doi:10.1080/17588921003632529) 24168333

[21] Fleming S. M. Weil R. S. Nagy Z. Dolan R. J. Rees G. 2010 Relating introspective accuracy to individual differences in brain structure. Science 329, 1541–1543 10.1126/science.1191883 (doi:10.1126/science.1191883) 20847276 PMC3173849

[22] Marti S. Sackur J. Sigman M. Dehaene S. 2010 Mapping introspection's blind spot: reconstruction of dual-task phenomenology using quantified introspection. Cognition 115, 303–313 10.1016/j.cognition.2010.01.003 (doi:10.1016/j.cognition.2010.01.003) 20129603

[23] Morsella E. Wilson L. E. Berger C. C. Honhongva M. Gazzaley A. Bargh J. A. 2009 Subjective aspects of cognitive control at different stages of processing. Atten. Percept. Psychophys. 71, 1807–1824 10.3758/APP.71.8.1807 (doi:10.3758/APP.71.8.1807) 19933564 PMC2784665

[24] Dienes Z. 2008 Subjective measures of unconscious knowledge. Prog. Brain Res. 168, 49–64 10.1016/S0079-6123(07)68005-4 (doi:10.1016/S0079-6123(07)68005-4) 18166385

[25] Schooler J. W. 2002 Re-representing consciousness: dissociations between experience and meta-consciousness. Trends Cogn. Sci. 6, 339–344 10.1016/S1364-6613(02)01949-6 (doi:10.1016/S1364-6613(02)01949-6) 12140084

[26] Schwartz B. Bacon E. 2008 Metacognitive neuroscience. In Handbook of memory and metamemory: essays in honor of Thomas O. Nelson (eds Dunlosky J. Bjork R.), pp. 355–371 New York, NY: Psychology Press

[27] Arbuckle T. 1969 Discrimination of item strength at time of presentation. J. Exp. Psych. 8, 126–131 10.1037/h0027455 (doi:10.1037/h0027455)

[28] Hart J. 1965 Memory and the feeling-of-knowing experience. J. Educ. Psychol. 56, 208–216 10.1037/h0022263 (doi:10.1037/h0022263) 5825050

[29] Brown A. S. 1991 A review of the tip-of-the-tongue experience. Psychol. Bull. 109, 204–223 10.1037/0033-2909.109.2.204 (doi:10.1037/0033-2909.109.2.204) 2034750

[30] Peirce C. S. Jastrow J. 1885 On small differences in sensation. Memoir. Natl Acad. Sci. 3, 73–83

[31] Vickers D. 1979 Decision processes in visual perception. New York, NY: Academic Press

[32] Eriksen C. W. 1960 Discrimination and learning without awareness: a methodological survey and evaluation. Psychol. Rev. 67, 279–300 10.1037/h0041622 (doi:10.1037/h0041622) 13697142

[33] Kunimoto C. 2001 Confidence and accuracy of near-threshold discrimination responses. Conscious. Cogn. 10, 294–340 10.1006/ccog.2000.0494 (doi:10.1006/ccog.2000.0494) 11697867

[34] Persaud N. McLeod P. Cowey A. 2007 Post-decision wagering objectively measures awareness. Nat. Neurosci. 10, 257–261 10.1038/nn1840 (doi:10.1038/nn1840) 17237774

[35] Hollard G. Massoni S. Vergnaud J. C. 2010 Subjective belief formation and elicitation rules: experimental evidence. Working paper no. 10088, Centre d'Economie, Université Panthéon-Sorbonne, Paris, France

[36] Becker G. M. DeGroot M. H. Marschak J. 1964 Measuring utility by a single-response sequential method. Behav. Sci. 9, 226–232 10.1002/bs.3830090304 (doi:10.1002/bs.3830090304) 5888778

[37] Dienes Z. Altmann G. Kwan L. 1995 Unconscious knowledge of artificial grammars is applied strategically. J. Exp. Psychol. Learn. Mem. Cogn. 21, 1322–1338 10.1037/0278-7393.21.5.1322 (doi:10.1037/0278-7393.21.5.1322)

[38] Macmillan N. Creelman C. 2005 Detection theory: a user's guide. New York, NY: Lawrence Erlbaum

[39] Green D. Swets J. 1966 Signal detection theory and psychophysics. New York, NY: Wiley

[40] Clarke F. Birdsall T. Tanner W. 1959 Two types of ROC curves and definition of parameters. J. Acoust. Soc. Am. 31, 629–630 10.1121/1.1907764 (doi:10.1121/1.1907764)

[41] Galvin S. J. Podd J. V. Drga V. Whitmore J. 2003 Type 2 tasks in the theory of signal detectability: discrimination between correct and incorrect decisions. Psychol. Bull. Rev. 10, 843–876 10.3758/BF03196546 (doi:10.3758/BF03196546) 15000533

[42] Evans S. Azzopardi P. 2007 Evaluation of a ‘bias-free’ measure of awareness. Spatial Vision 20, 61–77 10.1163/156856807779369742 (doi:10.1163/156856807779369742) 17357716

[43] Fleming S. M. Dolan R. J. 2010 Effects of loss aversion on post-decision wagering: implications for measures of awareness. Conscious. Cogn. 19, 352–363 10.1016/j.concog.2009.11.002 (doi:10.1016/j.concog.2009.11.002) 20005133 PMC2842936

[44] Maniscalco B. Lau H. In press A signal detection theoretic approach for estimating metacognitive sensitivity from confidence ratings. Conscious. Cogn. 10.1016/j.concog.2011.09.021 22071269

[45] Kersten D. Mamassian P. Yuille A. 2004 Object perception as Bayesian inference. Ann. Rev. Psychol. 55, 271–304 10.1146/annurev.psych.55.090902.142005 (doi:10.1146/annurev.psych.55.090902.142005) 14744217

[46] Kording K. 2007 Decision theory: what ‘should’ the nervous system do? Science 318, 606–610 10.1126/science.1142998 (doi:10.1126/science.1142998) 17962554

[47] Barthelmé S. Mamassian P. 2009 Evaluation of objective uncertainty in the visual system. PLoS Comput. Biol. 5, e1000504 (doi:10.1371/journal.pcbi.1000504 19750003 10.1371/journal.pcbi.1000504 PMC2730538

[48] Landy M. Goutcher R. Trommershäuser J. Mamassian P. 2007 Visual estimation under risk. J. Vis. 7, 4 10.1167/7.6.4 (doi:10.1167/7.6.4) 17685787 PMC2638507

[49] Whiteley L. Sahani M. 2008 Implicit knowledge of visual uncertainty guides decisions with asymmetric outcomes. J. Vis. 8, 2 10.1167/8.3.2 (doi:10.1167/8.3.2) 18484808 PMC2515365

[50] Trommershauser J. Maloney L. Landy M. 2003 Statistical decision theory and trade-offs in the control of motor response. Spatial Vision 16, 255–275 10.1163/156856803322467527 (doi:10.1163/156856803322467527) 12858951

[51] Smith J. D. Couchman J. J. Beran M. J. 2012 The highs and lows of theoretical interpretation in animal-metacognition research. Phil. Trans. R. Soc. B 367, 1297–1309 10.1098/rstb.2011.0366 (doi:10.1098/rstb.2011.0366) PMC3318761 22492748

[52] Kepecs A. Uchida N. Mainen H. A. Zariwala Z. F. 2008 Neural correlates, computation and behavioural impact of decision confidence. Nature 455, 227–231 10.1038/nature07200 (doi:10.1038/nature07200) 18690210

[53] Kiani R. Shadlen M. 2009 Representation of confidence associated with a decision by neurons in the parietal cortex. Science 324, 759–764 10.1126/science.1169405 (doi:10.1126/science.1169405) 19423820 PMC2738936

[54] Kepecs A. Mainen Z. F. 2012 A computational framework for the study of confidence in humans and animals. Phil. Trans. R. Soc. B 367, 1322–1337 10.1098/rstb.2012.0037 (doi:10.1098/rstb.2012.0037) PMC3318772 22492750

[55] Schwartz B. Metcalfe J. 1996 Methodological problems and pitfalls in the study of human metacognition. In Metacognition: knowing about knowing (eds Metcalfe J. Shimamura A.). Cambridge, MA: MIT Press

[56] Koriat A. 1997 Monitoring one's own knowledge during study: a cue-utilization approach to judgments of learning. J. Exp. Psych. Gen. 126, 349–370 10.1037/0096-3445.126.4.349 (doi:10.1037/0096-3445.126.4.349)

[57] Koriat A. 2007 Metacognition and consciousness. In The Cambridge handbook of consciousness (eds Zelazo P. D. Moscovitch M. Thompson E.), pp. 289–325, Cambridge, UK: Cambridge University Press

[58] Alter A. L. Oppenheimer D. M. 2009 Uniting the tribes of fluency to form a metacognitive nation. Personality Soc. Psychol. Rev. 13, 219–235 10.1177/1088868309341564 (doi:10.1177/1088868309341564) 19638628

[59] Busey T. A. Tunnicliff J. Loftus G. R. Loftus E. F. 2000 Accounts of the confidence-accuracy relation in recognition memory. Psychol. Bull. Rev. 7, 26–48 10.3758/BF03210724 (doi:10.3758/BF03210724) 10780019

[60] Koriat A. 1993 How do we know that we know? The accessibility model of the feeling of knowing. Psychol. Rev. 100, 609–639 10.1037/0033-295X.100.4.609 (doi:10.1037/0033-295X.100.4.609) 8255951

[61] Baranski J. V. Petrusic W. M. 1998 Probing the locus of confidence judgments: experiments on the time to determine confidence. J. Exp. Psych. Hum. Percept. Perform. 24, 929–945 10.1037/0096-1523.24.3.929 (doi:10.1037/0096-1523.24.3.929) 9627426

[62] Clifford C. Arabzadeh E. Harris J. 2008 Getting technical about awareness. Trends Cogn. Sci. 12, 54–58 10.1016/j.tics.2007.11.009 (doi:10.1016/j.tics.2007.11.009) 18178511

[63] Insabato A. Pannunzi M. Rolls E. T. Deco G. 2010 Confidence-related decision making. J. Neurophys. 104, 539–547 10.1152/jn.01068.2009 (doi:10.1152/jn.01068.2009) 20393062

[64] Rolls E. T. Grabenhorst F. Deco G. 2010 Choice, difficulty, and confidence in the brain. NeuroImage 53, 694–706 10.1016/j.neuroimage.2010.06.073 (doi:10.1016/j.neuroimage.2010.06.073) 20615471

[65] Higham P. A. Perfect T. J. Bruno D. 2009 Investigating strength and frequency effects in recognition memory using type-2 signal detection theory. J. Exp. Psychol. Learn. Mem. Cogn. 35, 57–80 10.1037/a0013865 (doi:10.1037/a0013865) 19210081

[66] Busey T. A. Arici A. 2009 On the role of individual items in recognition memory and metacognition: challenges for signal detection theory. J. Exp. Psychol. Learn. Mem. Cogn. 35, 1123–1136 10.1037/a0016646 (doi:10.1037/a0016646) 19686009

[67] Lau H. C. Passingham R. E. 2006 Relative blindsight in normal observers and the neural correlate of visual consciousness. Proc. Natl Acad. Sci. USA 103, 18 763–18 768 10.1073/pnas.0607716103 (doi:10.1073/pnas.0607716103) PMC1693736 17124173

[68] Wilimzig C. Tsuchiya N. Fahle M. Einhäuser W. Koch C. 2008 Spatial attention increases performance but not subjective confidence in a discrimination task. J. Vis. 8, 7.1–10 10.1167/8.5.7 (doi:10.1167/8.5.7) 18842078

[69] Izaute M. Bacon E. 2005 Specific effects of an amnesic drug: effect of lorazepam on study time allocation and on judgment of learning. Neuropsychopharmacology 30, 196–204 10.1038/sj.npp.1300564 (doi:10.1038/sj.npp.1300564) 15483562

[70] Song C. Kanai R. Fleming S. M. Weil R. S. Schwarzkopf D. S. Rees G. 2011 Relating inter-individual differences in metacognitive performance on different perceptual tasks. Conscious. Cogn. 20, 1787–1792 10.1016/j.concog.2010.12.011 (doi:10.1016/j.concog.2010.12.011) 21256051 PMC3203218

[71] Yeung N. Summerfield C. 2012 Metacognition in human decision-making: confidence and error monitoring. Phil. Trans. R. Soc. B 367, 1310–1321 10.1098/rstb.2011.0416 (doi:10.1098/rstb.2011.0416) PMC3318764 22492749

[72] Pleskac T. J. Busemeyer J. R. 2010 Two-stage dynamic signal detection: a theory of choice, decision time, and confidence. Psychol. Rev. 117, 864–901 10.1037/a0019737 (doi:10.1037/a0019737) 20658856

[73] Ratcliff R. Starns J. J. 2009 Modeling confidence and response time in recognition memory. Psychol. Rev. 116, 59–83 10.1037/a0014086 (doi:10.1037/a0014086) 19159148 PMC2693899

[74] Maniscalco B. Lau H. 2009 Evaluating signal detection models of perceptual decision confidence. Front. Syst. Neurosci. Conf. Abstract: Computational and systems neuroscience, 2009. 10.3389/conf.neuro.06.2009.03.335 (doi:10.3389/conf.neuro.06.2009.03.335)

[75] Gold J. Shadlen M. 2007 The neural basis of decision making. Ann. Rev. Neurosci. 30, 535–574 10.1146/annurev.neuro.29.051605.113038 (doi:10.1146/annurev.neuro.29.051605.113038) 17600525

[76] Middlebrooks P. G. Sommer M. A. 2011 Metacognition in monkeys during an oculomotor task. J. Exp. Psychol. Learn. Mem. Cogn. 37, 325–337 10.1037/a0021611 (doi:10.1037/a0021611) 21171807 PMC3649877

[77] Kruger J. Dunning D. 1999 Unskilled and unaware of it: how difficulties in recognizing one's own incompetence lead to inflated self-assessments. J. Pers. Soc. Psychol. 77, 1121–1134 10626367 10.1037//0022-3514.77.6.1121

[78] Kao Y. C. Davis E. S. Gabrieli J. D. E. 2005 Neural correlates of actual and predicted memory formation. Nat. Neurosci. 8, 1776–1783 10.1038/nn1595 (doi:10.1038/nn1595) 16286927

[79] Modirrousta M. Fellows L. K. 2008 Medial prefrontal cortex plays a critical and selective role in ‘feeling of knowing’ meta-memory judgments. Neuropsychologia 46, 2958–2965 10.1016/j.neuropsychologia.2008.06.011 (doi:10.1016/j.neuropsychologia.2008.06.011) 18606176

[80] Morgan M. Mason A. 1997 Blindsight in normal subjects? Nature 385, 401–402 10.1038/385401b0 (doi:10.1038/385401b0) 9009187

[81] Lau H. 2010 Are we studying consciousness yet? In Frontiers of consciousness: Chichele lectures (eds Weiskrantz L. Davies M.). Oxford, UK: Oxford University Press

[82] Shimamura A. P. 2000 Toward a cognitive neuroscience of metacognition. Conscious. Cogn. 9, 313–323 10.1006/ccog.2000.0450 (doi:10.1006/ccog.2000.0450) 10924251

[83] Hirst W. 1982 The amnesic syndrome: descriptions and explanations. Psychol. Bull. 91, 435–460 10.1037/0033-2909.91.3.435 (doi:10.1037/0033-2909.91.3.435) 7051076

[84] Zahr N. Kaufman K. 2011 Clinical and pathological features of alcohol-related brain damage. Nat. Rev. Neurol. 7, 284–294 10.1038/nrneurol.2011.42 (doi:10.1038/nrneurol.2011.42) 21487421 PMC8121189

[85] Shimamura A. P. Squire L. R. 1986 Memory and metamemory: a study of the feeling-of-knowing phenomenon in amnesic patients. J. Exp. Psychol. Learn. Mem. Cogn. 12, 452–460 10.1037/0278-7393.12.3.452 (doi:10.1037/0278-7393.12.3.452) 2942629

[86] Janowsky J. S. Shimamura A. P. Kritchevsky M. Squire L. R. 1989 Cognitive impairment following frontal lobe damage and its relevance to human amnesia. Behav. Neurosci. 103, 548 10.1037/0735-7044.103.3.548 (doi:10.1037/0735-7044.103.3.548) 2736069

[87] Pannu J. Kaszniak A. 2005 Metamemory experiments in neurological populations: a review. Neuropsychol. Rev. 15, 105–130 10.1007/s11065-005-7091-6 (doi:10.1007/s11065-005-7091-6) 16328731

[88] Schnyer D. M. Verfaellie M. Alexander M. P. LaFleche G. Nicholls L. Kaszniak A. W. 2004 A role for right medial prefontal cortex in accurate feeling-of-knowing judgements: evidence from patients with lesions to frontal cortex. Neuropsychologia 42, 957–966 10.1016/j.neuropsychologia.2003.11.020 (doi:10.1016/j.neuropsychologia.2003.11.020) 14998710

[89] Pannu J. Kaszniak A. Rapcsak S. 2005 Metamemory for faces following frontal lobe damage. J. Int. Neuropsychol. Soc. 11, 668–676 16248902 10.1017/S1355617705050873

[90] Kikyo H. Ohki K. Miyashita Y. 2002 Neural correlates for feeling-of-knowing. Neuron 36, 177–186 10.1016/S0896-6273(02)00939-X (doi:10.1016/S0896-6273(02)00939-X) 12367516

[91] Chua E. F. Schacter D. L. Rand-Giovannetti E. Sperling R. A. 2006 Understanding metamemory: neural correlates of the cognitive process and subjective level of confidence in recognition memory. NeuroImage 29, 1150–1160 10.1016/j.neuroimage.2005.09.058 (doi:10.1016/j.neuroimage.2005.09.058) 16303318

[92] Chua E. F. Schacter D. L. Sperling R. A. 2009 Neural correlates of metamemory: a comparison of feeling-of-knowing and retrospective confidence judgments. J. Cogn. Neurosci. 21, 1751–1765 10.1162/jocn.2009.21123 (doi:10.1162/jocn.2009.21123) 18823230 PMC2709699

[93] Kim H. Cabeza R. 2007 Trusting our memories: dissociating the neural correlates of confidence in veridical versus illusory memories. J. Neurosci. 27, 12190 10.1523/JNEUROSCI.3408-07.2007 (doi:10.1523/JNEUROSCI.3408-07.2007) 17989285 PMC6673246

[94] Moritz S. Gläscher J. Sommer T. Büchel C. Braus D. F. 2006 Neural correlates of memory confidence. NeuroImage 33, 1188–1193 10.1016/j.neuroimage.2006.08.003 (doi:10.1016/j.neuroimage.2006.08.003) 17029986

[95] Schnyer D. M. Nicholls L. Verfaellie M. 2005 The role of VMPC in metamemorial judgments of content retrievability. J. Cogn. Neurosci. 17, 832–846 10.1162/0898929053747694 (doi:10.1162/0898929053747694) 15904549 PMC1193651

[96] Yokoyama O. 2010 Right frontopolar cortex activity correlates with reliability of retrospective rating of confidence in short-term recognition memory performance. Neurosci. Res. 68, 199–206 10.1016/j.neures.2010.07.2041 (doi:10.1016/j.neures.2010.07.2041) 20688112

[97] Park H. J. Kim J. J. Lee S. K. Seok J. H. Chun J. Kim D. I. Lee J. D. 2008 Corpus callosal connection mapping using cortical gray matter parcellation and DT-MRI. Hum. Brain Mapping 29, 503–516 10.1002/hbm.20314 (doi:10.1002/hbm.20314) PMC6870924 17133394

[98] Kanai R. Rees G. 2011 The structural basis of inter-individual differences in human behaviour and cognition. Nat. Rev. Neurosci. 12, 231–242 10.1038/nrn3000 (doi:10.1038/nrn3000) 21407245

[99] Ramnani N. Owen A. M. 2004 Anterior prefrontal cortex: insights into function from anatomy and neuroimaging. Nat. Rev. Neurosci. 5, 184–194 10.1038/nrn1343 (doi:10.1038/nrn1343) 14976518

[100] Semendeferi K. Teffer K. Buxhoeveden D. P. Park M. S. Bludau S. Amunts K. Travis K. Buckwalter J. 2011 Spatial organization of neurons in the frontal pole sets humans apart from Great Apes. Cereb. Cortex 21, 1485–1497 10.1093/cercor/bhq191 (doi:10.1093/cercor/bhq191) 21098620

[101] Simons J. S. Henson R. N. A. Gilbert S. J. Fletcher P. C. 2008 Separable forms of reality monitoring supported by anterior prefrontal cortex. J. Cogn. Neurosci. 20, 447–457 10.1162/jocn.2008.20036 (doi:10.1162/jocn.2008.20036) 18004946 PMC2292823

[102] Yoshida W. Ishii S. 2006 Resolution of uncertainty in prefrontal cortex. Neuron 50, 781–789 10.1016/j.neuron.2006.05.006 (doi:10.1016/j.neuron.2006.05.006) 16731515

[103] Gilbert S. J. Spengler S. Simons J. S. Frith C. D. Burgess P. W. 2006 Differential functions of lateral and medial rostral prefrontal cortex (area 10) revealed by brain-behavior associations. Cereb. Cortex 16, 1783–1789 10.1093/cercor/bhj113 (doi:10.1093/cercor/bhj113) 16421331

[104] Buda M. Fornito A. Bergström Z. M. Simons J. S. 2011 A specific brain structural basis for individual differences in reality monitoring. J. Neurosci. 31, 14 308–14 313 10.1523/JNEUROSCI.3595-11.2011 (doi:10.1523/JNEUROSCI.3595-11.2011) PMC3190297 21976516

[105] Curtis C. D'Esposito M. 2003 Persistent activity in the prefrontal cortex during working memory. Trends Cogn. Sci. 7, 415–423 10.1016/S1364-6613(03)00197-9 (doi:10.1016/S1364-6613(03)00197-9) 12963473

[106] Sakai K. Rowe J. B. Passingham R. E. 2002 Active maintenance in prefrontal area 46 creates distractor-resistant memory. Nat. Neurosci. 5, 479–484 10.1038/nn846 (doi:10.1038/nn846) 11953754

[107] Scheperjans F. Hermann K. Eickhoff S. B. Amunts K. Schleicher A. Zilles K. 2008 Observer-independent cytoarchitectonic mapping of the human superior parietal cortex. Cereb. Cortex 18, 846–867 10.1093/cercor/bhm116 (doi:10.1093/cercor/bhm116) 17644831

[108] John J. P. Yashavantha B. S. Gado M. Veena R. Jain S. Ravishankar S. Csernansky J. G. 2007 A proposal for MRI-based parcellation of the frontal pole. Brain Struct. Funct. 212, 245–253 10.1007/s00429-007-0157-x (doi:10.1007/s00429-007-0157-x) 17929054

[109] Fleming S. M. Huijgen J. Dolan R. J. Submitted Prefrontal mechanisms for awareness of task performance.

[110] Smith R. Keramatian K. Christoff K. 2007 Localizing the rostrolateral prefrontal cortex at the individual level. NeuroImage 36, 1387–1396 10.1016/j.neuroimage.2007.04.032 (doi:10.1016/j.neuroimage.2007.04.032) 17532648

[111] Thompson W. B. Mason S. E. 1996 Instability of individual differences in the association between confidence judgments and memory performance. Mem. Cogn. 24, 226–234 10.3758/BF03200883 (doi:10.3758/BF03200883) 8881325

[112] Kelemen W. L. Frost P. J. Weaver C. A. 2000 Individual differences in metacognition: evidence against a general metacognitive ability. Mem. Cogn. 28, 92–107 10.3758/BF03211579 (doi:10.3758/BF03211579) 10714142

[113] Fleck M. S. Daselaar S. M. Dobbins I. G. Cabeza R. 2006 Role of prefrontal and anterior cingulate regions in decision-making processes shared by memory and nonmemory tasks. Cereb. Cortex 16, 1623–1630 10.1093/cercor/bhj097 (doi:10.1093/cercor/bhj097) 16400154

[114] Sharot T. Riccardi A. Raio C. 2007 Neural mechanisms mediating optimism bias. Nature 450, 102–105 10.1038/nature06280 (doi:10.1038/nature06280) 17960136

[115] Hassabis D. Maguire E. 2007 Deconstructing episodic memory with construction. Trends Cogn. Sci. 11, 299–306 10.1016/j.tics.2007.05.001 (doi:10.1016/j.tics.2007.05.001) 17548229

[116] Botvinick M. M. Braver T. S. Barch D. M. Carter C. S. Cohen J. D. 2001 Conflict monitoring and cognitive control. Psychol. Rev. 108, 624–652 10.1037/0033-295X.108.3.624 (doi:10.1037/0033-295X.108.3.624) 11488380

[117] Ridderinkhof K. R. Ullsperger M. Crone E. A. Nieuwenhuis S. 2004 The role of the medial frontal cortex in cognitive control. Science 306, 443–447 10.1126/science.1100301 (doi:10.1126/science.1100301) 15486290

[118] Ullsperger M. Harsay H. A. Wessel J. R. Ridderinkhof K. R. 2010 Conscious perception of errors and its relation to the anterior insula. Brain Struct. Funct. 214, 629–643 10.1007/s00429-010-0261-1 (doi:10.1007/s00429-010-0261-1) 20512371 PMC2886909

[119] MacDonald A. W. Cohen J. D. Stenger V. A. Carter C. S. 2000 Dissociating the role of the dorsolateral prefrontal and anterior cingulate cortex in cognitive control. Science 288, 1835–1838 10.1126/science.288.5472.1835 (doi:10.1126/science.288.5472.1835) 10846167

[120] Kerns J. G. Cohen J. D. MacDonald A. W. Cho R. Y. Stenger V. A. Carter C. S. 2004 Anterior cingulate conflict monitoring and adjustments in control. Science 303, 1023–1026 10.1126/science.1089910 (doi:10.1126/science.1089910) 14963333

[121] Fernandez-Duque D. Baird J. A. Posner M. I. 2000 Executive attention and metacognitive regulation. Conscious. Cogn. 9, 288–307 10.1006/ccog.2000.0447 (doi:10.1006/ccog.2000.0447) 10924249

[122] Arango-Muñoz S. 2010 Two levels of metacognition. Philosophia 39, 71–82 10.1007/s11406-010-9279-0 (doi:10.1007/s11406-010-9279-0)

[123] Carruthers P. 2009 How we know our own minds: the relationship between mindreading and metacognition. Behav. Brain Sci. 32, 121–138 10.1017/S0140525X09000545 (doi:10.1017/S0140525X09000545) 19386144

[124] Proust J. 2007 Metacognition and metarepresentation: is a self-directed theory of mind a precondition for metacognition? Syntheses 159, 271–295 10.1007/s11229-007-9208-3 (doi:10.1007/s11229-007-9208-3)

[125] Evans J. 2008 Dual-processing accounts of reasoning, judgment, and social cognition. Annu. Rev. Psychol. 59, 255–278 10.1146/annurev.psych.59.103006.093629 (doi:10.1146/annurev.psych.59.103006.093629) 18154502

[126] Shea N. Heyes C. 2010 Metamemory as evidence of animal consciousness: the type that does the trick. Biol. Philos. 25, 95–110 10.1007/s10539-009-9171-0 (doi:10.1007/s10539-009-9171-0) 20234826 PMC2837231

[127] Logan G. D. Crump M. J. C. 2010 Cognitive illusions of authorship reveal hierarchical error detection in skilled typists. Science 330, 683–686 10.1126/science.1190483 (doi:10.1126/science.1190483) 21030660

[128] Wenke D. Fleming S. M. Haggard P. 2010 Subliminal priming of actions influences sense of control over effects of action. Cognition 115, 26–38 10.1016/j.cognition.2009.10.016 (doi:10.1016/j.cognition.2009.10.016) 19945697

[129] Medalla M. Barbas H. 2010 Anterior cingulate synapses in prefrontal areas 10 and 46 suggest differential influence in cognitive control. J. Neurosci. 30, 16 068–16 081 10.1523/JNEUROSCI.1773-10.2010 (doi:10.1523/JNEUROSCI.1773-10.2010) PMC3064955 21123554

[130] Naccache L. Dehaene S. Cohen L. Habert M.-O. Guichart-Gomez E. Galanaud D. Willer J.-C. 2005 Effortless control: executive attention and conscious feeling of mental effort are dissociable. Neuropsychologia 43, 1318–1328 10.1016/j.neuropsychologia.2004.11.024 (doi:10.1016/j.neuropsychologia.2004.11.024) 15949516

[131] McGuire J. T. Botvinick M. M. 2010 Prefrontal cortex, cognitive control, and the registration of decision costs. Proc. Natl Acad. Sci. USA 107, 7922–7926 10.1073/pnas.0910662107 (doi:10.1073/pnas.0910662107) 20385798 PMC2867898

[132] Nieuwenhuis S. Ridderinkhof K. R. Blom J. Band G. P. H. Kok A. 2001 Error-related brain potentials are differentially related to awareness of response errors: evidence from an antisaccade task. Psychophysiology 38, 752–760 10.1111/1469-8986.3850752 (doi:10.1111/1469-8986.3850752) 11577898

[133] Freedman D. J. Assad J. A. 2011 A proposed common neural mechanism for categorization and perceptual decisions. Nat. Neurosci. 14, 143–146 10.1038/nn.2740 (doi:10.1038/nn.2740) 21270782

[134] Heekeren H. Marrett S. Ruff D. Bandettini P. Ungerleider L. 2006 Involvement of human left dorsolateral prefrontal cortex in perceptual decision making is independent of response modality. Proc. Natl Acad. Sci. USA 103, 10 023–10 028 10.1073/pnas.0603949103 (doi:10.1073/pnas.0603949103) 16785427 PMC1479865

[135] Ho T. C. Brown S. Serences J. T. 2009 Domain general mechanisms of perceptual decision making in human cortex. J. Neurosci. 29, 8675–8687 10.1523/JNEUROSCI.5984-08.2009 (doi:10.1523/JNEUROSCI.5984-08.2009) 19587274 PMC2719543

[136] David A. S. Bedford N. Wiffen B. Gilleen J. 2012 Failures of metacognition and lack of insight in neuropsychiatric disorders. Phil. Trans. R. Soc. B 367, 1379–1390 10.1098/rstb.2012.0002 (doi:10.1098/rstb.2012.0002) PMC3318769 22492754

